# Clinical Effectiveness of Primary and Secondary Headache Treatment by Transcranial Direct Current Stimulation

**DOI:** 10.3389/fneur.2013.00025

**Published:** 2013-03-21

**Authors:** Dmitry Pinchuk, Olga Pinchuk, Konstantin Sirbiladze, Olga Shugar

**Affiliations:** ^1^Department of Neurology, Municipal Center for Medical Rehabilitation of Children with Psychoneurological DisordersSaint Petersburg, Russia; ^2^Neurolife SALausanne, Switzerland; ^3^Department of Neurology, Rodnik Medical CenterPerm, Russia

**Keywords:** tDCS, treatment of headache, tension-type headache, chronic post-traumatic headache, migraine

## Abstract

The clinical effectiveness of primary and secondary headache treatment by transcranial direct current stimulation (tDCS) with various locations of stimulating electrodes on the scalp was analyzed retrospectively. The results of the treatment were analyzed in 90 patients aged from 19 to 54 years (48 patients had migraine without aura, 32 – frequent episodic tension-type HAs, 10 – chronic tension-type HAs) and in 44 adolescents aged 11–16 years with chronic post-traumatic HAs after a mild head injury. Clinical effectiveness of tDCS with 70–150 μA current for 30–45 min via 6.25 cm^2^ stimulating electrodes is comparable to that of modern pharmacological drugs, with no negative side effects. The obtained result has been maintained on average from 5 to 9 months. It has been demonstrated that effectiveness depends on localization of stimulating electrodes used for different types of HAs.

## Introduction

Over the past decade, transcranial direct current stimulation (tDCS) has been more widely used as a mean of neuromodulation for targeted alteration of CNS structures excitability not only in neurophysiological (Wassermann et al., 2008; Stagg and Nitsche, 2011), but also in clinical studies for treatment of depressions (Nitsche et al., 2009), stroke consequences (Schlaug et al., 2008), pain syndrome (Fregni et al., 2006; Zaghi et al., 2009), and other effects.

Stimulation by low-intensity direct current, or so-called galvanization, is not new. Galvanizations had been widely used worldwide from the beginning of nineteenth till the beginning of twentieth century; it gradually gave place to impulse stimulations with more predicable results (Lolas, 1977). Fundamental physiological basis of one of galvanization types, called tDCS in Europe and the USA, or transcranial micropolarization (TCMP) in Russia, including safety issues and general principles of clinical use, have already been stated in the 1970s and 1980s in the works of Institute of Experimental Medicine (Saint Petersburg) (Vartanian et al., 1981). We started to use tDCS in a clinical practice since 1988. We published first reports on its successful use in children with cerebral palsy as far back as in 1994 (Bogdanov et al., 1994). The method of brain galvanization (transcranial direct current stimulation, one of the types of which is the tDCS) is officially approved in the Russian Federation (Order No. 1440 of 21.12.1984 by the Ministry of Health of the USSR, and Orders of 12.07.2004 and 27.12.2011 No. 1664n “On approval of the nomenclature of medical services” by the Ministry of Health and Social Development of the Russian Federation) subject to the following rules: voltage up to 50 V, current up to 10 mA, current density for adults 0.05–0.3 mA/cm^2^, for children of 5–12 months not more than 0.03 mA/cm^2^, with age the current density increases up to 0.08 mA/cm^2^ (in children 15–16 years of age), exposure time from 10 to 40 min.

Nowadays, tDCS is a routine technique used for various pathologies at the Saint Petersburg Municipal Center for Medical Rehabilitation of Children with Psychoneurological Disorders (Pinchuk, 2007). Since 1988 till 2011, more than 1400 children and adolescents aged from 9 months to 17 years and more than 350 adult patients with various nervous system diseases have undergone tDCS treatment in the Center. tDCS procedures appeared to be also effective in the treatment of headaches (HA). Based on results obtained we tried to identify the possible mechanisms underlying the clinical effectiveness tDCS in the treatment of HA.

We are aware that this work is as yet incomplete but taking into consideration the importance of the problem, great number of patients with HA, combined with the high degree of effectiveness of the technique and almost complete absence of undesirable effects, we consider advisable to inform specialists working in this field of our experience in using tDCS technique for HA treatment.

### Aim

The objective of this work is to provide a retrospective analysis of the results of the treatment of patients with HA by means of tDCS with various localizations of stimulating electrodes on the scalp and to define possible mechanisms of action at these positions for various types of HA.

## Materials and Methods

The retrospective study was utilized to evaluate the results of tDCS treatment in patients with different types of primary and secondary HAs. We did not conduct trials using simulated tDCS, nor did we use control groups or compare the efficacy of alternative treatment; we confined ourselves to comparing our results with the published data of other authors on the efficacy of the pharmacological treatment of patients with HAs. The data of clinical studies, which were included in the standard tDCS protocol used in our Center, have been analyzed. Protocol includes reports by the patients themselves (parental reports for children), results of electroencephalographic (EEG) investigation (an obligatory procedure when selecting patients for tDCS treatment) and brief Lüscher test with eight colors (Sobchik, 2001). In specific cases, MRI and CT were used, as well as ultrasound examination of brain vessels and vessels of vertebrobasilar basin was conducted in order to exclude symptomatic HA.

In addition for adult patients a visual pain scale (Numerical Rating Scale – NRS) from 0 to 10 (Belova and Shepetova, 2002), Beck Depression Inventory (Belova and Shepetova, 2002), and Spielberger’s State-Trait Anxiety Inventory, adapted by Khanin (1976) for local conditions, were used. Among 165 persons receiving tDCS sessions for HA, patients were selected whose HA symptoms corresponded to diagnostic criteria of International Classification of Headache Disorders (ICHD-II) (Headache Classification Subcommittee of the International Headache Society, 2004), which allowed us to classify them as the certain type of primary HA – migraine without aura (ICHD-II 2004 code 1.1), frequent episodic tension-type HA (FETTH) (ICHD-II 2.2), chronic tension-type HA (CTTH) (ICHD-II 2.3), and secondary HA – chronic post-traumatic HA related to a mild head injury (CPTH) (ICHD-II 5.2.2).

We analyzed the results of HA treatment in 90 adults aged from 19 to 54 (mean age 29.4 ± 12.8 years), 71% (64 persons) were women and 29% (26 persons) were men, and also treatment results of 44 adolescents (38 boys and 6 girls aged from 11 to 16; mean age 13.6 ± 2.5 years).

In the analyzed groups we used three basic localizations of stimulating electrodes on the scalp out of 17 used during tDCS.

For the first electrode position (1EP), an anode was placed over the frontal pole (medial edge of the electrode was situated at the boundary of the interhemispheric fissure) of the hemisphere subdominant in motor skills; a cathode was located at the ipsilateral mastoid process.

For the second electrode position (2EP), an anode was placed in the center of the forehead, 1.5 cm above the nasal bridge, at the projection of the interhemispheric fissure; a cathode was placed 2 cm higher the mastoid process of the hemisphere subdominant in motor skills.

For the third electrode position (3EP), an anode was placed in the center of the frontal pole of subdominant hemisphere; a cathode was placed 2 cm above the ipsilateral mastoid process.

Stimulation time was 30–45 min both in children and adults; current intensity ranged from 60 to 90 μA for children to 100–150 μA for adults; electrodes were made of medical conductive rubber and were placed in 6.25 cm^2^ saline-soaked multilayer flannel cases. The current densities we use (0.001–0.024 mA/cm^2^) almost do not differ from those used in majority of studies on tDCS (0.029–0.066 mA/cm^2^) (Bastani and Jaberzadeh, 2012), and they are in the range permitted by the Ministry of Health of the USSR (now the Russian Federation) when performing transcranial stimulation with galvanic (direct) current (0.01–0.2 mA/cm^2^). By the way, these figures are almost the same as the parameters of current density which were used at the end of nineteenth to twentieth centuries (0.01–0.3 mA/cm^2^). The time of stimulation we use (30–45 min) is somewhat longer than the one traditionally used in procedures of tDCS (10–20 min). However, in several studies (Schlaug and Renga, 2008) longer time is used (up to 30 min). Lengthening the time of exposure leads to greater charge of electricity than during the traditional use; tDCS – 0.09–0.12 C/cm^2^, where 1 C is the amount of electric charge transported in 1 s by a steady current of 1 A (Stagg and Nitsche, 2011), in our studies these values range from 0.19 to 0.26 C/cm^2^. Although, there are works where these values are higher than the ones we used. So in the study of Schlaug and Renga (2008) the maximum total charge was 2.4 C/cm^2^. However, in this case the values are much lower than the acceptable safety regulations (up to 200 C/cm^2^) (Yuen et al., 1981). The number of procedures for a course varied from five to nine with 4–7 days interval. Stabilization of HA level was a criterion for treatment course completion. Treatment was conducted in compliance with modern generally accepted standards of biomedical ethics.

The main parameter of treatment effectiveness included a decrease in number of days with HA per month by 50% or more, compared with baseline prior to the treatment; secondary parameters included HA intensity and duration, amount of analgesics used, depression, and anxiety scale parameters. Parameters prior to and after the treatment were compared between the groups using the Wilcoxon non-parametric test for paired comparisons of dependent samples and also the non-parametric Spearmen correlations (Statistica, StatSoft, version 6.1).

## Results

The group with migraine consisted of 48 patients aged 19–54 (32.9 ± 10.3) years: 79% (38 persons) were women, 21% (10 persons) – men; the mean duration of disease was 11.7 ± 10.1 years. Characteristic of patients is presented in Table 1.

**Table 1:** **Results of tDCS treatment of various HA types (in analyzed groups in general)**.

	Migraine (48 persons)		FETTH (32 persons)		CPTH (44 persons)		CTTH (10 persons)	
	Before	After	Before	After	Before	After	Before	After
NRS score	7.52 ± 2.38	3.92 ± 2.84***	5.52 ± 1.94	3.26 ± 2.51**	5.11 ± 1.6	2.11 ± 1.54***	5.76 ± 2.53	3.62 ± 2.44
Number of days with HA per month	4.71 ± 1.53	1.44 ± 2.27***	8.83 ± 3.51	4.97 ± 4.67**	10.32 ± 6.48	4.11 ± 2.18***	21.77 ± 5.34	17.43 ± 7.66
Attack duration (h)	18.32 ± 9.21	5.23 ± 3.42***	6.73 ± 4.82	4.75 ± 3.82	4.57 ± 3.76	2.45 ± 1.66***	7.71 ± 3.25	4.83 ± 3.78
Level of depression (score) according to Beck Inventory	14.22 ± 6.54	10.21 ± 4.11**	17.22 ± 6.54	13.28 ± 3.71*	–	–	12.54 ± 5.63	10.81 ± 6.75
Level of trait anxiety (score) (by Spielberger)	44.85 ± 9.75	40.15 ± 11.35*	48.14 ± 11.61	43.67 ± 7.34	–	–	32.95 ± 5.61	30.48 ± 5.37
Level of state anxiety (score) (by Spielberger)	42.77 ± 8.21	36.43 ± 15.1**	51.17 ± 9.17	42.04 ± 11.18***	–	–	36.02 ± 7.11	29.28 ± 6.75*
Amount of analgesics taken (tablets/month)	12.07 ± 8.62	5.27 ± 3.48***	10.16 ± 4.23	7.43 ± 3.31*	–	–	16.10 ± 6.62	13.11 ± 4.25

All values are provided in *M* ± σ format. Values prior to and after the treatment significantly differed according to the paired Wilcoxon test for dependent samples at the level (*p < 0.05, **p < 0.01, ***p < 0.001).

Unspecific diffuse changes in bioelectrical activity (BA) of varying degrees were revealed on ECGs of almost all patients with migraine. Even though the picture of EEGs of patients with migraine is polymorphic, two groups can be identified.

Electroencephalographic changes typical for dysfunction of brain stem oral departments with activation of its structures were observed in the first group (Niedermeyer and Lopes da Silva, 1999). Decreased ά-rhythm amplitude and index, up to a complete disappearance of the rhythm, smoothing of regional differences, domination of low-amplitude polymorphic slow activity distorted by flashes of regular θ-rhythm in frontal leads and bilateral synchronic flashes of β-rhythm in temporal and frontal leads were observed. The range of reaction of adopting the photostimulation rhythm was increased (from 2 to 24 Hz). Virtually no epileptiform activity and focal changes were observed on EEGs of patients in this subgroup.

On EEGs of the second group the following was observed: increased amplitude of ά-rhythm (more than 120 μV), increase of its index to 70–80%, ά-rhythm frequency instability, its improper distribution (it was registered in frontal leads), and distortion of ά-rhythm by flashes of regular θ-rhythm (5–6 Hz) in frontal and central leads. The range of adopting the rhythm of photostimulation increased from 2 to 22 Hz.

Epileptiform activity was registered in some patients (single slow spikes, reduced epi-complexes, and paroxysmal activity when conducting stress tests). These alterations are observed at diencephalic structures dysfunction (Niedermeyer and Lopes da Silva, 1999).

Patients with the first type of EEG composed 35% and about 40% with the second type; approximately 10% of EEGs of patients with migraine were normal, 15% of the patients had a mixed EEG type.

The group of patients with FETTH consisted of 32 patients aged from 24 to 49 (33.6 ± 12.3): 23 women (72%) and 9 men (28%). In 65% (21 persons) HA was associated with pericranial tenderness (ICHD-II 2004 code 2.2.1), 11 patients had no pericranial tenderness (code 2.2.2).

An ά-rhythm, with significantly decreased index and amplitude, insufficiently modulated and desynchronized, was registered on EEGs of majority of the patients with FETTH. Slow activity manifested as low-amplitude polymorphic slow activity. Considerable elevation of excitability processes was observed, which was reflected in generalized adopting of reaction on photostimulation in the wide range of frequencies (from 2 to 24 Hz), and also in ά-, θ-, and β-waves flashes in frontal leads.

The group of patients with CTTH consisted of 10 women aged from 31 to 52 (35.2 ± 11.8) with HA during 17–26 days a month (19.77 ± 5.34 on average). Pain pattern corresponded to ICHD-II (Headache Classification Subcommittee of the International Headache Society, 2004) diagnostic criteria of CTTH except for the article on medication overuse.

Unspecific alterations of brain BA were revealed in most cases in EEGs of patients with CTTH: decreased amplitude and irregularity of ά-rhythm, domination of rapid waves, sharpened and slow oscillations, smoothing of regional differences. EEGs are normal or marginal in 4 out of 10 patients. Background paroxysmal activity as single flashes of spike waves was observed only in one female patient.

A group of patients with CPTH related to a mild head injury consisted of adolescents undergoing treatment in the Center for cognitive disorders, asthenia, anxiety, vegetative disturbances occurred in 6–9 months after a mild brain concussion. A CTTH of moderate intensity usually occurred in the afternoon.

Elevated spectrum intensity of ά-rhythm in frontal departments of the brain and its decrease in caudal departments, combined with reduction of ά-rhythm amplitude, were observed on EEGs. In five patients (12%), a regular θ-rhythm has been observed in frontal and central leads. Data on effectiveness of tDCS usage in analyzed groups in general is presented in Table 1, and with various location of stimulating electrodes – in Table 2.

**Table 2:** **Effectiveness of treatment with various localizations of stimulating electrodes on the scalp for various HA types**.

Positions of stimulating electrodes on the scalp	Migraine (48 persons)			FETTH (32 persons)			CPTH (44 persons)		CTTH (10 persons)	
	1	2	3	1	2	3	1	2	1	3
Number of patients in the group	33	13	2	18	10	4	38	6	3	7
HA absence for at least 4.5 months	9 (27%)	3 (23%)	–	4 (22%)	3 (30%)	–	20 (52%)		–	–
Reduction of the number of days with HA at least by 50% compared with baseline for at least 4.5 months	17 (51%)	8 (62%)	–	6 (33%)	5 (50%)	2 (50%)	11 (29%)	2 (33%)	–	2 (29%)
Total improvement	26 (78%)	11 (85%)	–	10 (55%)	8 (50%)	2 (50%)	33 (81%)		–	–
No effect	6 (19%)	2 (15%)	2 (100%)	7 (39%)	1 (10%)	2 (50%)	7 (19%)	4 (66%)	3 (100%)	5 (71%)
Worsening of condition	1 (3%)	–	–	1 (6%)	1 (10%)	–	–		–	

According to patients’ self-reports, tDCS, when using the 1EP, has led to fast HA relief (already during the first two to three sessions). This localization has been more effective at CPTH and migraine (more than in 85 and 78%, respectively), at FETTH (52%), at CTTH (only in two patients out of seven, i.e., 29%). The 1EP was more effective in patients with dominating increased tonus of parasympathetic nervous system (according to Lüscher test – so-called criterion of vegetative balance) (Belova and Shepetova, 2002).

With migraine, the number of attacks decreased; if developed, they were shorter and less intensive. The pattern of HAs changed: during the attack, HA was dull instead of acute and disappeared rather fast. Duration of pain attack reduced from 9–24 to 3–8 h. The number of vegetative manifestations (flushes, feeling hot, sweating, nausea, etc.) sharply decreased. Amount of analgesics, relieving the attack, decreased from 2–6 to 1–2 tablets.

Out of 33 patients with migraine who received tDCS at the 1EP, HA became worse only in one 42-year-old female patient after the second tDCS session; as a result this the patient refused to continue treatment. HA intensity in this patient decreased to baseline in 2 weeks after treatment termination and remained at that level throughout 3 months of follow-up.

Subjective self-assessment of HA level in the group of patients with migraine when using the 1EP has significantly decreased by NRS score (on average by 3.01; from 7.32 ± 1.54 to 4.31 ± 1.82 after the treatment) according to the Wilcoxon test (*p* = 0.0024). Depression level significantly decreased according to Beck Inventory (from 13.81 ± 5.19 to 10.01 ± 3.94, *p* = 0.044).

Not only the level of state anxiety but also the level of trait anxiety statistically significantly decreased according to Spielberger’s Inventory. Significant Spearman correlations (*p* < 0.05) of NRS subjective pain assessment were observed for the level of state anxiety only, but not for the trait one. Duration of obtained sustained clinical alterations, as a rule, was at least 5 months (6–8 months on average). The longest recorded result was 2.5 years.

After tDCS course in patients with CPTH, along with marked reduction in HA level, the following was observed: significant reduction of asthenic syndrome manifestations (reduced tiredness and irritancy, normalization of sleep), reduction, and in some patients almost complete disappearance of vegetative lability symptoms. In 52% of patients with CPTH, after tDCS course HA completely disappeared for at least 4.5 months; in 28% of patients the number of days with HA decreased by at least 50% from baseline for at least 4.5 months; tDCS had no effect in 20% of patients. Subjective assessment of HA level in patients with CPTH decreased by 3.5 points on average in the group (from 5.4 ± 1.8 prior to the treatment to 1.9 ± 1.2 after tDCS course, *p* = 0.0006).

After tDCS course, in patients with FETTH the feeling of head compression reduced significantly or completely disappeared, the number of days without HA increased, amount of analgesics being taken reduced. Level of pain in patients with FETTH when using the 1EP reduced by 1.88 points on average in the group (4.92 ± 2.89 prior to the treatment, 3.04 ± 2.82 after the treatment, *p* = 0.062). Positive clinical effect was obtained mainly due to the subgroup of patients with TTH combined with muscular tenderness of pericranial muscles. Out of 18 patients with TTH, receiving tDCS in the 1EP, TTH combined with pericranial tenderness was observed in 13 patients. Out of these 13 patients, HAs completely disappeared in 4 patients for a period longer than 4.5 months; in 6 patients, the number of days with HA reduced by at least 50% compared with baseline for a period longer than 4.5 months; only in 3 patients no effect was observed. tDCS clinical effectiveness assessed by the decrease of days with HA in total is 76.9% in the subgroup of patients with FETTH combined with pericranial tenderness. According to NRS, reduction of HA intensity is significant (from 5.05 ± 3.01 prior to the treatment to 2.87 ± 2.11, *p* = 0.0429). Reduction of HA intensity significantly (*p* < 0.05) correlated (by Spearman) both with decrease of depression level by Beck Inventory and with decrease of state anxiety level by Spielberger’s Inventory. In all five patients with FETTH not associated with pericranial tenderness, tDCS with the 1EP was ineffective. Treatment course was terminated after the third procedure in a 35-years old female patient due to increasing of HAs accompanied with vegetative manifestations (dizziness, nausea). In a week after treatment termination, HA stabilized at the former level.

Obtained positive effect in patients with FETTH has preserved for at least 6 months (8–10 months on average); the longest recorded positive result was 18 months.

Neither decreased number of days with HA nor reduced HA was revealed in three patients with CTTH with the 1EP used; although no intensification of HA was either observed.

The 2EP was more effective at migraine with moderate pain attacks and with dominating moderately increased tonus of sympathetic autonomic nervous system (ANS). Out of 13 patients with migraine, receiving tDCS sessions, the shift of vegetative balance toward domination of sympathetic ANS was observed in 10 patients according to Lüscher test, and positive clinical response (complete disappearance or reduction of days with HA by 50% compared with baseline) was obtained in all of them. Reduced intensity of HAs by NRS from 7.78 ± 1.68 to 5.23 ± 1.19 (*p* = 0.002) was observed. Out of three patients with migraine, who had a shift of vegetative balance toward domination of parasympathetic ANS, reduced HA was observed in only one patient.

The 2EP was effective in some patients with FETTH, mainly at TTH not combined with pericranial tenderness. Out of 10 patients with TTH, receiving tDCS treatment with the 2EP, there were 6 patients with TTH not combined with pericranial tenderness. At that, clinical improvement was observed in five of them; all three patients, whose HAs disappeared for at least 4.5 months, had TTH not combined with pericranial tenderness. Improvement was registered only in two out of four patients with FETTH combined with pericranial tenderness: no effect was observed in one patient, and one patient terminated treatment due to intensification of HA.

The 2EP was used in six patients with CPTH. Treatment was effective only in two girls aged 13 and 13.5 years. In four boys (aged from 9 to 11 years) treatment was ineffective. “Mildness” of treatment effect, which progressively increased at every session and stabilized by sessions five to seven, is an advantage of using the 2EP.

Out of all positions used, the 3EP was the most effective at CTTH (although it is rather conditional, as some improvement was observed only in two out of six patients).

## Discussion

Although TTH and migraine are traditionally considered to be different diseases, a significant clinical effectiveness of the same electrode localization (the 1EP) at tDCS both in patients with migraine and in patients with TTH rather indicates common pathophysiological mechanisms in these HA types. We tend to agree with D. Greenberg et al. (2005), who consider migraine and TTH to be two opposite poles of a single clinical spectrum. Psychosocial stress is believed to be a provoking factor for TTH, and personality traits (high incidence of anxiodepressive and somatoform disorders) predispose to development of cephalgia (Karakulova, 2006). However, these factors and, in particular, anxiodepressive syndrome of varying degrees are revealed in almost 60% of migraine cases (Gusev et al., 1999; Osipova and Levin, 2006). NRS HA intensity level correlated significantly with state anxiety parameters according to Spielberger’s Inventory and Beck Depression Inventory in our study both in patients with migraine and in patients with TTH.

Electroencephalography in our patients with migraine (the first type of EEG) is similar although not identical to EEG of patients with FETTH. It is interesting that this type of EEG (decrease of ά-rhythm index and amplitude, domination of irregular ά-, β-, and θ-oscillations on EEG, and increased range of photostimulation adoption reaction) is typical for patients with anxiodepressive syndrome (Volynkina and Suvorov, 1981). The latter also confirms common pathophysiological mechanisms of migraine (patients with the first EEG type) and TTH in some cases. This explains a positive clinical effect when using the same electrode position (the 1EP) during tDCS both in patients with migraine and in patients with FETTH.

Apparently, changes in balance of frontal cortex activity, its shift toward activation of left hemisphere frontal cortex, determining the positive emotional background, is one of the reasons for improvement following tDCS with the 1EP, taking into consideration anxiodepressive component in HA clinical picture (Deglin and Nikolaenko, 1975; Heller, 1993). tDCS inhibitory regimen (exposure more than 35 min) (Pinchuk, 2007) applied on the structures of the right orbitofrontal cortex resulted in a decrease in its activity and in reciprocal activation of the left hemisphere released of the right hemisphere inhibiting influence. This leads to a marked mood improvement, reduced anxiety, more adequate assessment, and response to environment and to regression of concurrent psychovegetative symptoms and reduction of HA intensity.

Transcranial direct current stimulation with the 1EP has predominantly caused an increase of sympathetic ANS activity which manifested in a feeling of energy surge, in decreased sleeping period, however, without feeling fatigued, in increased libido (prior to that it was decreased on average). Insignificant transitory systolic arterial pressure increase (by 5–10 mm Hg) was observed in some patients directly after tDCS. An increased level of sympathetic ANS activity is supposed to result in rebalancing of parasympathetic and sympathetic ANS. Due to this fact, among others, this localization in patients with symptoms of increased parasympathetic ANS activity is the most effective one. However, abrupt HA reduction during tDCS cannot be explained only by improvement of patient’s psycho-emotional condition. Alterations in brainstem reticular formation (RF) and, particularly, in mesencephalic RF during tDCS seem to play an important role in observed effects. Activating locations with similar low threshold for activation and inhibition reactions as in non-specific RF structures of mesencephalon and thalamus are revealed in different cortical areas (Penfield and Jasper, 1954; Andreyeva et al., 1979). Our results also suggest the possibility of targeted alteration in RF functional state by influencing the modulating cortical areas (Pinchuk, 2007).

Used localization of stimulating electrodes allowed us to influence not only the frontal pole, but also (although to a lesser extent) the mediobasal areas of the frontal lobes, from which the most powerful system of corticofugal fibers goes toward the RF. Precisely by influencing these areas we can lower or increase RF and thalamus activity to an optimal level (Tsirkin and Trukhina, 2001).

Electroencephalographic pattern in patients with migraine and tension-type HA indicates either significantly increased excitability of cortical elements or reduced cortical tonus, which in both cases can indicate disturbances in functional correlations between the cortex and mesencephalic RF in HA. Normalization of these correlations seems to occur during tDCS.

Activation of mesencephalic RF is supposed to be the main treatment factor when using tDCS in patients with CPTH after a mild brain injury. The leading cause of these HAs is a reduction of RF activating influence, leading to disorder of reticulo-cortico-subcortical neurodynamics (Kryzhanovsky, 1986; Callaghan and Abu-Arafeh, 2001). Thus, the 1EP, providing the most prominent RF activation effect, is the most effective in such patients (in 85% of patients receiving tDCS sessions).

Complaints between attacks point to a chronic dysfunction of hypothalamic system both in patients with migraine and in patients with TTH. Low tolerance for different provoking factors (low “migraine threshold”), also stress and vasodilator factors indicates the same. Improved hypothalamus functioning during tDCS manifests in normalization of patients’ vegetative status (improvement of psycho-emotional condition, reduced sweating and chilliness in the arms and legs, normalization of blood pressure and gastrointestinal functions, in particular, normalization of stool in patients with long-lasting and persistent constipations etc.). Sometimes, especially during the first sessions, when stimulation regimens are chosen inadequately, symptoms registered in experiments with direct hypothalamus stimulation (“gargantuan appetite,” nausea, increased salivation, intestinal cramps etc.) are observed directly during tDCS procedure (for 15–20 min). These symptoms were most observed when using the 2EP, less frequently – in the 1EP and 3EP. The above-observations suggest that hypothalamus condition also changes during tDCS. It is still unclear whether a hypothalamus condition changes due to direct tDCS impact on the cortical areas with corticofugal projections on the hypothalamus (possibly, during tDCS with the 2EP), or hypothalamus activation is secondary to RF activation. The latter is more likely for the 1EP and 3EP, it can be explained by the powerful system of reticulo-hypothalamic relations.

Besides the above-mentioned mechanisms, cathode’s position also plays an important role in reducing a HA. Intensity of emotional feelings regardless of their type (positive or negative) and, respectively, the level of vegetative reactions providing such emotional condition depend on the state of the right post-temporal area (Aftanas and Varlamov, 2007). Exactly this area was inhibited by a cathode in tDCS using the 1EP and 2EP on the scalp.

Clinical effectiveness of tDCS at HA treatment (excluding CTTH) is comparable to an effectiveness of traditional pharmacological drugs and to such non-traditional types of treatment as biofeedback technique and chiropractic manipulations (Andrasik, 2010; Haag, 2010; Bryans et al., 2011). At the same time, therapeutic effect was more consistent and prolonged than during medication therapy with almost no side effects; it makes tDCS a promising treatment option for HA of various etiologies.

## Conflict of Interest Statement

The authors declare that the research was conducted in the absence of any commercial or financial relationships that could be construed as a potential conflict of interest.

## Abbreviations

1EP, first electrode position; 2EP, second electrode position; 3EP, third electrode position; ANS, autonomic nervous system; CPTH, chronic post-traumatic headache related to a mild head injury; CTTH, chronic tension-type headache; EEG, electroencephalography; FETTH, frequent episodic tension-type headache; HA, headaches; ICHD-II, international classification of headache disorders (2004); NRS, numerical rating scale; RF, reticular formation; tDCS, transcranial direct current stimulation; TTH, tension-type headache.

## References

[B1] Aftanas L. I. Varlamov A. A. (2007). Effects of alexithymia on the activity of the anterior and posterior areas of the cortex of the right hemisphere in positive and negative emotional activation. Neurosci. Behav. Physiol. 37, 67–73 10.1007/s11055-007-0151-z 17180321

[B2] Andrasik F. (2010). Biofeedback in headache: an overview of approaches and evidence. Cleve. Clin. J. Med. 77, 72–76 10.3949/ccjm.77.s3.13 20622082

[B3] Andreyeva V. N. Kratin Y. G. Sotnichenko T. S. (1979). Low-threshold mechanisms and cortical activation pathways. Neurosci. Behav. Physiol. 9, 387–394 10.1007/BF01185063 492508

[B4] Bastani A. Jaberzadeh S. (2012). Does anodal transcranial direct current stimulation enhance excitability of the motor cortex and motor function in healthy individuals and subjects with stroke: a systematic review and meta-analysis. Clin. Neurophysiol. 123, 644–657 10.1016/j.clinph.2011.08.029 21978654

[B5] Belova A. Shepetova O. (2002). Scales, Tests, and Questionnaires in Medical Rehabilitation: Guide for Physicians and Research Fellows. Moscow: Antidor. [in Russian]

[B6] Bogdanov O. V. Pinchuk D. Yu. Pisar’kova E. V. (1994). The use of the method of transcranial micropolarization to decrease the severity hyperkineses in patients with infantile cerebral palsy. Neurosci. Behav. Physiol. 24, 442–445 10.1007/BF02359800 7838369

[B7] Bryans R. Descarreaux M. Duranleau M. Marcoux H. Potter B. Ruegg R. (2011). Evidence-based guidelines for the chiropractic treatment of adults with headache. J. Manipulative Physiol. Ther. 34, 274–289 10.1016/j.jmpt.2011.04.008 21640251

[B8] Callaghan M. Abu-Arafeh I. (2001). Chronic posttraumatic headache in children and adolescents. Dev. Med. Child Neurol. 43, 819–822 10.1111/j.1469-8749.2001.tb00169.x 11769268

[B9] Deglin V. L. Nikolaenko N. N. (1975). Role of the dominant hemisphere in the regulation of emotion states. Hum. Physiol. 1, 16 [in Russian].

[B10] Fregni F. Gimenes R. Valle A. C. Ferreira M. J. Rocha R. R. Natalle L. (2006). A randomized, sham-controlled, proof of principle study of transcranial direct current stimulation for the treatment of pain in fibromyalgia. Arthritis Rheum. 54, 3988–3998 10.1002/art.22195 17133529

[B11] Greenberg D. A. Aminoff M. J. Simon R. P. (2005). Clinical Neurology, 6th Edn New York: McGraw-Hill Medical

[B12] Gusev Ye. I. Burd G. S. Nikiforov A. S. (1999). Neurological Symptoms, Syndromes, Symptom Complexes, and Diseases. Moscow: Meditsina. [in Russian]

[B13] Haag G. (2010). Headache and medication overuse: are clinical case series appropriate to reveal differential risks of different medications? Expert Opin. Drug Saf. 9, 397–406 10.1517/14740331003598857 20166835

[B14] Headache Classification Subcommittee of the International Headache Society. (2004). The international classification of headache disorders: 2nd edition. Cephalalgia 24, 1–52 10.1111/j.1468-2982.2003.00824.x 14979299

[B15] Heller W. (1993). Neuropsychological mechanisms of individual differences in emotion, personality and arousal. Neuropsychology 7, 476–489 10.1037/0894-4105.7.4.476

[B16] Karakulova Iu. V. (2006). Pathogenetic mechanisms of tension headache development. Zh. Nevrol. Psikhiatr. Im. S. S. Korsakova 106, 52–56 [in Russian]. 16921720

[B17] Khanin Yu. L. (1976). Brief Handbook for Application of ChD Spilberger’s Personality and Situative Anxiety Score. Moscow: Meditsina. [in Russian]

[B18] Kryzhanovsky G. N. (1986). Central Nervous System Pathology. A New Approach. London: Springer

[B19] Lolas F. (1977). Brain polarization: behavioral and therapeutic effects. Biol. Psychiatry 12, 37–47 300033

[B20] Niedermeyer E. Lopes da Silva F. (ed.). (1999). Electroencephalography: Basic Principles, Clinical Applications and Related Fields. Baltimore, MD: Williams & Wilkins

[B21] Nitsche M. A. Boggio P. S. Fregni F. Pascual-Leone A. (2009). Treatment of depression with transcranial direct current stimulation (tDCS): a review. Exp. Neurol. 219, 14–19 10.1016/j.expneurol.2009.03.038 19348793

[B22] Osipova V. V. Levin Ya. I. (2006). Migraine and “sleep-wake” cycle. Zh. Nevrol. Psikhiatr. Im. S. S. Korsakova 106, 9–15 [in Russian]. 16768218

[B23] Penfield W. Jasper H. (1954). Epilepsy and the Functional Anatomy of the Human Brain. Boston: Little, Brown

[B24] Pinchuk D. Yu. (2007). Transcranial Micropolarization of Brain. Clinical Picture. Physiology. 20 Years of Clinical Experience. St. Petersburg: Chelovek. [in Russian]

[B25] Schlaug G. Renga V. (2008). Transcranial direct current stimulation: a noninvasive tool to facilitate stroke recovery. Expert Rev. Med. Devices 5, 759–768 10.1586/17434440.5.6.759 19025351 PMC3176333

[B26] Schlaug G. Renga V. Nair D. (2008). Transcranial direct current stimulation in stroke recovery. Arch. Neurol. 65, 1571–1576 10.1001/archneur.65.12.1571 19064743 PMC2779259

[B27] Sobchik L. N. (2001). MTSV – A Method of Color Choices. The Modified Eight-Test Luscher. Practical Guide. St. Petersburg: Pervoye Sentyabrya. [in Russian]

[B28] Stagg C. J. Nitsche M. A. (2011). Physiological basis of transcranial direct current stimulation. Neuroscientist 17, 37–53 10.1177/1073858410386614 21343407

[B29] Tsirkin V. I. Trukhina S. I. (2001). The Physiological Basis of Mental Activity and Behavior. Russia: Novgorod. [in Russian]

[B30] Vartanian G. A. Galdinov G. V. Akimova I. M. (1981). Organization and Modulation of Memory Processes. Leningrad: USSR Academy of Medical Sciences. [in Russian]

[B31] Volynkina G. Yu. Suvorov N. F. (1981). Neurophysiological structure of human emotional states. Leningrad: Nauka. [in Russian]

[B32] Wassermann E. M. Epstein C. M. Ziemann U. Walsh V. Paus T. Lisanby S. H. (2008). The Oxford Handbook of Transcranial Stimulation. New York: Oxford University Press Inc

[B33] Yuen T. G. Agnew W. F. Bullara L. A. Jacques S. McCreery D. B. (1981). Histological evaluation of neural damage from electrical stimulation: considerations for the selection of parameters for clinical application. Neurosurgery 9, 292–299 10.1097/00006123-198109000-00013 7301072

[B34] Zaghi S. Heine N. Fregni F. (2009). Brain stimulation for the treatment of pain: a review of costs, clinical effects, and mechanisms of treatment for three different central neuromodulatory approaches. J. Pain Manag. 2, 339–352 20585474 PMC2888303

